# Associations between artificial sweetener intake from cereals, coffee, and tea and the risk of type 2 diabetes mellitus: A genetic correlation, mediation, and mendelian randomization analysis

**DOI:** 10.1371/journal.pone.0287496

**Published:** 2024-02-07

**Authors:** Youqian Zhang, Zitian Tang, Yong Shi, Lin Li

**Affiliations:** 1 Department of Endocrinology, The First Affiliated Hospital of Yangtze University, Jingzhou, Hubei, China; 2 Department of Law, Yangtze University, Jingzhou, Hubei, China; 3 Department of Medicine, Yangtze University, Jingzhou, Hubei, China; University of Hawai’i at Manoa College of Tropical Agriculture and Human Resources, UNITED STATES

## Abstract

**Background:**

Previous studies have emphasized the association between the intake of artificial sweeteners (AS) and type 2 diabetes mellitus (T2DM), but the causative relationship remains ambiguous.

**Methods:**

This study employed univariate Mendelian randomization (MR) analysis to assess the causal link between AS intake from various sources and T2DM. Linkage disequilibrium score (LDSC) regression was used to evaluate the correlation between phenotypes. Multivariate and mediation MR were applied to investigate confounding factors and mediating effects. Data on AS intake from different sources (N = 64,949) were sourced from the UK Biobank, while T2DM data were derived from the DIAbetes Genetics Replication And Meta-analysis.The primary method adopted was inverse variance weighted (IVW), complemented by three validation techniques. Additionally, a series of sensitivity analyses were performed to evaluate pleiotropy and heterogeneity.

**Results:**

LDSC analysis unveiled a significant genetic correlation between AS intake from different sources and T2DM (r_g_ range: -0.006 to 0.15, all *P* < 0.05). After correction by the false discovery rate (FDR), the primary IVW method indicated that AS intake in coffee was a risk factor for T2DM (OR = 1.265, 95% CI: 1.035–1.545, *P* = 0.021, *P*_*FDR*_ = 0.042). Further multivariable and mediation MR analyses pinpointed high density lipoprotein-cholesterol (HDL-C) as mediating a portion of this causal relationship. In reverse MR analysis, significant evidence suggested a positive correlation between T2DM and AS intake in coffee (β = 0.013, 95% CI: 0.004–0.022, *P* = 0.004, *P*_*FDR*_ = 0.012), cereal (β = 0.007, 95% CI: 0.002–0.012, *P* = 0.004, *P*_*FDR*_ = 0.012), and tea (β = 0.009, 95% CI: 0.001–0.017, *P* = 0.036, *P*_*FDR*_ = 0.049). No other causal associations were identified (*P* > 0.05, *P*_*FDR*_ > 0.05).

**Conclusion:**

The MR analysis has established a causal relationship between AS intake in coffee and T2DM. The mediation by HDL-C emphasizes potential metabolic pathways underpinning these relationships

## Introduction

Diabetes mellitus (DM) represents a significant and pressing global health concern [1], with type 2 diabetes mellitus (T2DM) constituting approximately 90% of all diabetes cases worldwide [2]. The World Health Organization (WHO) estimates that there are currently over 422 million diabetics globally and that there will be 629 million by the year 2045 [3, 4]. Notably, the prevalence of diabetes has experienced an upward trajectory in developing nations, including China and Pakistan, leading to considerable direct and indirect financial strain on society [5]. Consequently, the identification of novel modifiable risk factors for T2DM is imperative for informing clinical management strategies and mitigating the onset and progression of the disease.

As lifestyles change, the demand for sweet treats is gradually increasing. Artificial sweeteners (AS), as low-calorie and sugar-free alternatives, have gained popularity as sugar substitutes to decrease caloric intake [6]. The most popular AS include aspartame, saccharin, acesulfame potassium, and sucralose [7], commonly used in foods and beverages such as cereals [8], coffee [9], and tea [10] to satisfy the demand for sweetness. Current research has identified associations between AS and T2DM; however, findings from observational studies in this domain often exhibit inconsistencies. Certain investigations have reported a 3% elevated relative risk of T2DM per additional daily serving of AS [11–14], while others have demonstrated that the intake of artificially sweetened beverages, when compared to water, is associated with a 21% rise in T2DM incidence [15]. Moreover, no correlation between AS and T2DM has been shown in other studies [16, 17]. Despite the widespread use of AS in the daily diet and their popularity among people with T2DM, there is no consensus on a causal relationship between them and diabetes due to inconsistent research findings.

Previous research encountered challenges in establishing a definitive causal relationship between exposure factors and outcome variables, largely attributable to complexities stemming from confounding variables and reverse causation. Given the constraints of observational studies in ascertaining causality with certainty, alternative approaches such as Mendelian randomization (MR) in the realm of genetic research can prove to be invaluable. Experiments that employ MR utilize genetic variations, ascertained through genome-wide association analyses, as instrumental variables (IVs). These IVs help in gauging the causal relationship between environmental exposure and the desired outcome. Under certain conditions, this technique allows for drawing causal inferences by using genetic variants as surrogates for environmental exposure [18]. Conceived as a natural randomized controlled trial, MR is based on Mendelian inheritance laws that allocate parental alleles to their offspring. This approach offers a more robust degree of evidence and a diminished vulnerability to confounding factors. In contrast to observational epidemiological research, MR presents a higher caliber of evidence. This study aims to employ univariate MR (UVMR), multivariate MR (MVMR), mediation MR, and linkage disequilibrium score (LDSC) regression to investigate the relationship between intake of AS from various sources and T2DM, further delving into the mediating roles of five confounding factors.

## Materials and methods

### Study design

**Fig 1:**
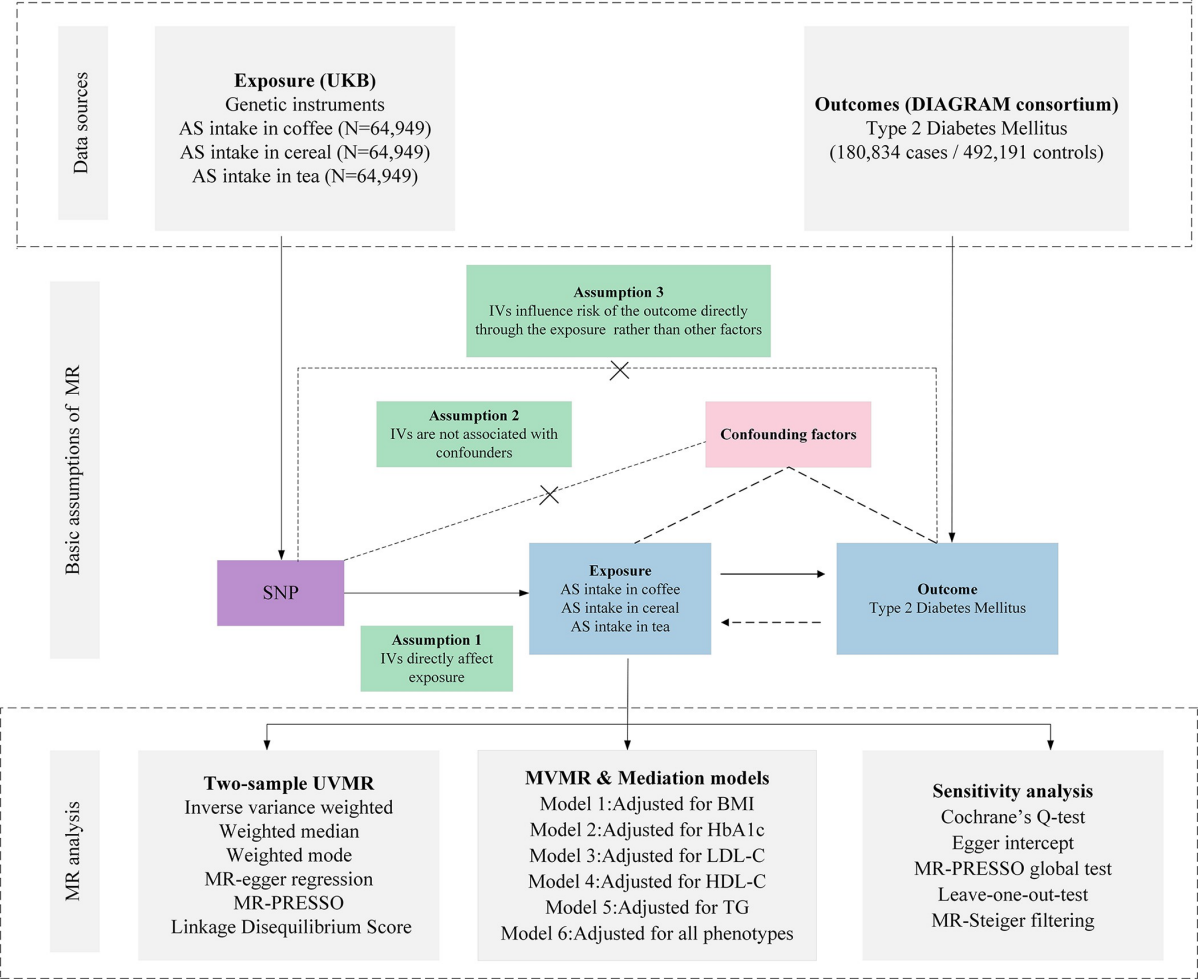
Overview of research design and analysis strategy. Overview of the research design. Exposures come from UKB, with outcomes including Type 2 diabetes mellitus. The MR framework is based on three fundamental MR assumptions, with MVMR analyses adjusting for five mediating factors for positive results. MVMR, Multivariate MR; UVMR, Univariate MR; BMI: Body Mass Index; SNP, Single Nucleotide Polymorphism; MR-PRESSO, MR Pleiotropy Residual Sum and Outlier; HbA1c, Glycated Hemoglobin A1c; LDL-C, Low Density Lipoprotein Cholesterol; HDL-C, High Density Lipoprotein Cholesterol; TG, Triglyceride; DIAGRAM, DIAbetes Genetics Replication And Meta-analysis; UKB, UK Biobank; AS, Artificial sweetener.

The foundational datasets for this study were procured from genome-wide association studies (GWAS). Each GWAS study included obtained the necessary approvals from their respective institutional review boards. As this study involves a secondary analysis of publicly available data, no additional ethical permissions were required. IVs for the exposure were identified based on three critical criteria: (i) the selected genetic variant, serving as the IV, should have a strong association with the exposure; (ii) this variant should not be associated with any known confounders; and (iii) the effect of the variant on the outcome should be solely through the exposure, negating any alternative pathways [19]. The MR approach is detailed in **Fig 1**, while the summary statistics from the data sources are presented in **Table 1**.

**Table 1:** Detailed information of data sources.

Explore or Outcome	Ref	Ieu id	Consortium	Ancestry	Participants
**Phenotypes**					
AS intake in coffee	36402876	ukb-b-1338	UKB	European	64,949 individuals
AS intake in cereal	36402876	ukb-b-3143	UKB	European	64,949 individuals
AS intake in tea	36402876	ukb-b-5867	UKB	European	64,949 individuals
T2DM	35551307	NA	DIAGRAM	European	180,834 cases / 492,191 controls
**Adjustment of the model**					
LDL-C	24097068	ieu-a-300	GLGC	96% European	173,082 individuals
HDL-C	24097068	ieu-a-299	GLGC	96% European	187,167 individuals
TG	24097068	ieu-a-302	GLGC	96% European	177,861 individuals
HbA1c	20858683	ieu-b-104	MAGIC	European	46,368 individuals
BMI	30124842	ieu-b-40	GIANT	European	681,275 individuals

DIAGRAM, DIAbetes Genetics Replication And Meta-analysis; T2DM, type 2 Diabetes Mellitus; UKB, UK Biobank; BMI, body mass index; GIANT, Genetic Investigation of Anthropometric Traits; GLGC, Global Lipids Genetics Consortium; MAGIC, Meta-Analyses of Glucose and Insulin-related traits Consortium; LDL-C, Low Density Lipoprotein Cholesterol; HDL-C, high density lipoprotein-cholesterol; TG, triglyceride; HbA1c, glycated hemoglobin A1c; Ref, reference(Pubmed id); AS, artificial sweetener.

### Selection of genetic instrumental variables

The summary-level GWAS data for AS intake in coffee/tea/cereal were all sourced from the UK Biobank (UKB) [20], encompassing 64,949 European individuals. This information was collected using questionnaires where participants detailed the amount of AS (for example, Canderel) they added to their daily coffee or tea/infusion on a per-drink basis. Additionally, those who reported consuming cereal or porridge the previous day specified the quantity of sweetener added per bowl. To ensure the accuracy of MR analyses, we adhered to stringent criteria for single nucleotide polymorphism (SNPs) selection: (i) SNPs selected as IVs must show an association with the defined exposure at a genome-wide significance level (*P* < 5×10^−8^). Given the absence of genome-wide significant SNPs for exposure, we applied a relaxed threshold of 5×10^−6^ to capture more SNPs for these phenotypes [21]. (ii) Chosen SNPs were further filtered to ensure no associations with potential confounders and to preserve independence among them, thereby mitigating potential biases from linkage disequilibrium (r^2^ < 0.001, clumping distance = 10,000 kb). (iii) The efficacy of the selected SNPs as IVs was validated using F-statistics (F = beta^2^/se^2^; beta for SNP-exposure association; variance (se)), assessing the possibility of weak instrumental variables [22]. A high F-statistic indicates robust instrumental strength, and our criteria required all SNPs to have an F-statistic above 10. (iv) To enhance the reliability of our results, we applied MR-Steiger filtering, which systematically excludes variants more correlated with outcomes than exposures [23]. (v) In cases where an SNP is absent from the outcome dataset, we utilized the SNiPa online platform (http://snipa.helmholtz-muenchen.de/snipa3/), based on European population genotype data from the 1000 Genomes Project’s Phase 3, to locate the missing SNP and identify a proxy SNP with strong linkage disequilibrium (criteria set at r^2^ > 0.8) to the original SNP. (vi) The effect of the SNP on the exposure and its effect on the outcome should align with the same allele.

### Source of outcome phenotypes

The summary-level GWAS meta-analysis for T2DM integrated 22 cohorts, sourced from the AMP-T2D Knowledge Portal and the DIAbetes Genetics Replication And Meta-analysis (DIAGRAM) consortium [24]. T2DM is defined by ICD-10 codes and includes 180,834 cases and 492,191 controls from European populations.

### Data sources for possible mediators

We further obtained genetic associations for Body Mass Index (BMI) from the Genetic Investigation of Anthropometric Traits (GIANT) consortium [25], Glycated Hemoglobin A1c (HbA1c) from Meta-Analyses of Glucose and Insulin-related traits Consortium (MAGIC) [26], triglycerides (TG), Low-Density Lipoprotein Cholesterol (LDL-C) and High-Density Lipoprotein Cholesterol (HDL-C) from Global Lipids Genetics Consortium (GLGC) [27].

## Statistical analyses

### Primary MR analysis

Within the UVMR framework, individual IVs were evaluated using the Wald ratio test. For scenarios involving multiple IVs, the multiplicative random-effects inverse-variance-weighted (IVW) approach was utilized, with ancillary use of the MR-Egger and weighted median methodologies. In the IVW approach, the weight accorded aligns directly with the Wald ratio estimation and inversely with the variance of each respective SNP [28]. IVW offers dependable outcomes when all genetic variants are appropriate; however, the weighted median is optimal when a majority are deemed inappropriate, and MR-Egger is reserved for complete invalidity [29]. Moreover, we utilized the constrained maximum likelihood (CML) method for our analysis. This technique allows for the combined estimation across multiple genetic variants while considering possible confounders and genetic heterogeneity. Using CML ensures enhanced precision and reliability in our estimates, especially in scenarios involving an abundance of genetic variants and potential confounding variables [30]. Consideration for multiple comparisons was made through the false discovery rate (FDR), with a post-adjustment *P*-value < 0.05 indicating a discernible causal association. Situations where a raw *P*-value was < 0.05, yet exceeded 0.05 post-FDR adjustment, were considered indicative rather than conclusive.

Within the scope of the Mediation MR and MVMR analysis, and recognizing potential confounders such as BMI, HbA1c, TG, LDL-C, and HDL-C in the exposure-outcome trajectory, we employed MVMR to discern the inherent causal relationship. The initial MVMR postulation centers on the association of genetic variations with specific exposures, while subsequent postulates align with UVMR standards [31]. An assessment was conducted to quantify mediated effects. Commencing with MR, effect estimates correlated to exposures were ascertained via the IVW approach. Subsequently, MVMR was utilized to quantify the influence of the aforementioned mediating factors on outcomes. The product of the two estimates for each outcome yielded the exposure’s indirect influence. The ratio of the mediated to the total effect facilitated an understanding of each mediator’s contribution to the cumulative effect.

### Genetic correlation analysis

Linkage disequilibrium score (LDSC) regression, when tailored to summary-level GWAS datasets, emerges as a sophisticated technique to interrogate genetic correlations in intricate diseases or varied phenotypic manifestations. With precision, this methodology segregates authentic polygenic associations from potential confounders, such as cryptic relatedness or population stratification [32]. A statistically and quantitatively significant genetic correlation implies that the aggregate phenotype correlation extends beyond mere environmental confounders [32]. For detailed investigations into genetic correlations between specific exposures and corresponding phenotypic outcomes, the LDSC platform is available at https://github.com/bulik/ldsc.

### Sensitivity analysis

Within the UVMR structure, we executed a series of tests to validate the analytical integrity. The Cochran’s Q test was utilized to measure heterogeneity among the chosen genetic markers, recognizing a *P*-value under 0.05 as indicative of significant variances between the SNPs under examination [33]. The MR-Egger regression was employed to explore the potential of directional pleiotropy in the MR framework [34]. An intercept *P*-value below 0.05 in the MR-Egger regression indicates a considerable directional pleiotropy, albeit the method may pose limitations in accuracy [35]. The MR Pleiotropy Residual Sum and Outlier (MR-PRESSO) was invoked to pinpoint outliers and assess horizontal pleiotropy, deeming a global *P*-value under 0.05 as significant [36]. This technique’s exclusion of outliers fine-tunes the correction process. Additionally, the leave-one-out method was incorporated to evaluate the influence of individual SNPs on the overall results [37].

The R^2^ value was computed using the formula 2×MAF×(1-MAF)×beta^2^, with MAF representing the minor allele frequency of each instrumental SNP. Summation of these values yielded the coefficient pivotal for power computation [38]. We derived the statistical power utilizing tools available on the mRnd website [39] (https://shiny.cnsgenomics.com/mRnd/).

## Results

### Genetic instrument selection and genetic correlation between phenotypes

The study reports F statistics exceeding 20 for all IVs, signifying a robust reduction of bias from weak instruments. The SNPs selected as IVs ranged from 14 to 176, accounting for an explained variance of 0.09% to 28.47%, and the power statistics obtained ranged from 6% to 100% **(S1 Table)**.

LDSC analysis revealed a significant genetic correlation between AS intake in coffee (r_g_: 0.384, *P* = 1.19×10^−8^), AS intake in tea (r_g_: 0.263, *P* = 3.70×10^−3^), and AS intake in cereal (r_g_: 0.932, *P* = 8.12×10^−7^)with T2DM. The SNP-based heritability (h^2^) was 1.42% (coffee), 0.24% (tea), 1.43% (cereal) and 5.71% (T2DM).

### Association of genetically predicted exposure with outcome

**Fig 2:**
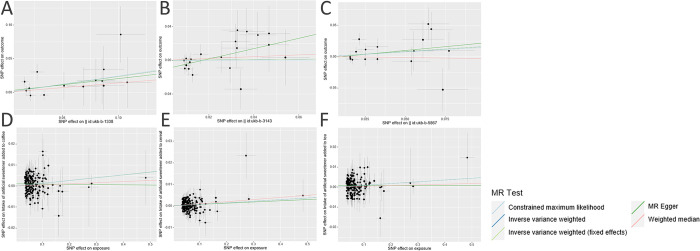
Genetic associations with AS intake from different sources (horizontal axis, standard deviation units) and with T2DM (vertical axis, log odds ratios) at a genome- wide level of significance. (A) AS intake in coffee on T2DM (B) AS intake in tea on T2DM (C) AS intake in cereal on T2DM (D) T2DM on AS intake in coffee (E) T2DM on AS intake in tea (F) T2DM on AS intake in cereal. Horizontal and vertical lines represent 95% confidence intervals for the genetic associations. Horizontal and vertical lines represent 95% confidence intervals for the genetic associations. AS, Artificial sweetener; T2DM, type 2 diabetes mellitus.

**Fig 3:**
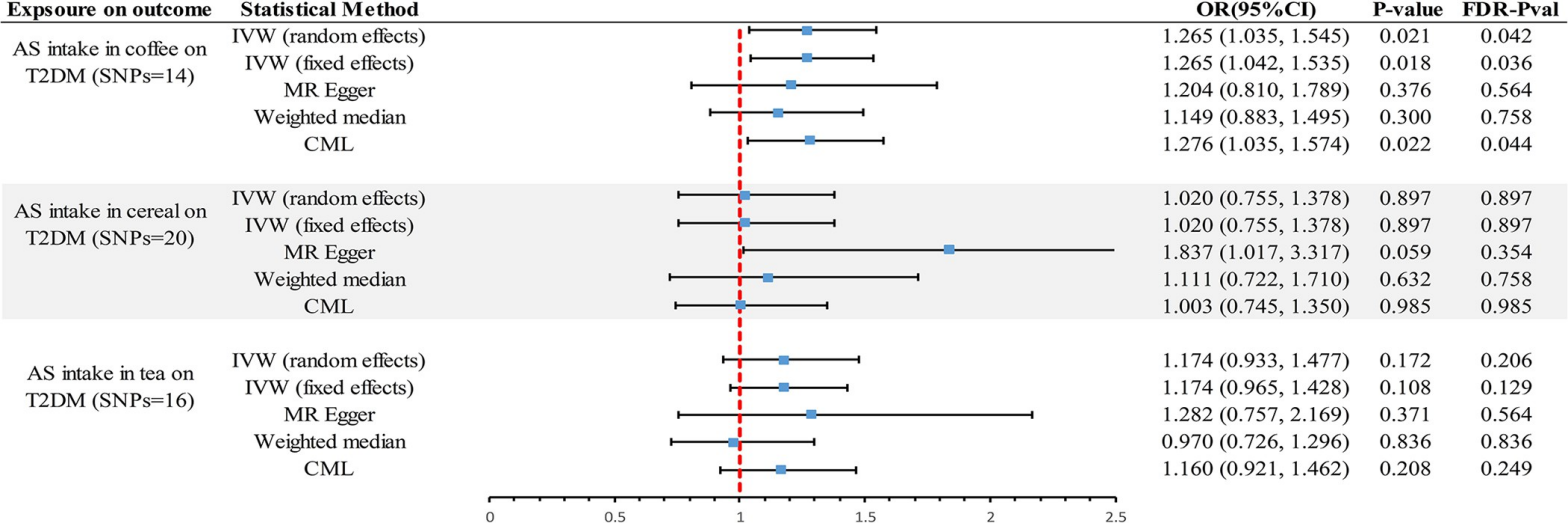
Genetically predicted causal associations of intake of different sources of AS on T2DM were assessed by different methods. IVW, Inverse-Variance-Weighted; FDR, False Discovery Rate; OR, odds ratio; CI, confidence interval; AS, artificial sweetener; T2DM, type 2 diabetes; CML, constrained maximum likelihood.

The scatter plot provides a clear visualization of all positive results **(Fig 2)**. After applying the FDR correction for multiple comparisons, our primary IVW analysis demonstrated strong causal evidence **(Fig 3)**. Specifically, for every standard deviation (SD) increase in genetically predicted AS intake in coffee, the risk of T2DM increased by 26% (OR = 1.265, 95% CI 1.035~1.545, *P* = 0.021, *P*_*FDR*_ = 0.042). Complementary methods IVW-fixed effects (OR = 1.265, 95% CI 1.042~1.535, *P* = 0.018, *P*_*FDR*_ = 0.036)and CML (OR = 1.276, 95% CI 1.035~1.574, *P* = 0.022, *P*_*FDR*_ = 0.044) provide evidence of consistency. With an OR of 1.265, we possess 98% statistical power to detect an association between AS intake in coffee and T2DM.Furthermore, no causal association was identified between AS intake in cereal and T2DM (OR = 1.020, 95% CI 0.755~1.378, *P* = 0.897, *P*_*FDR*_ = 0.897), and similarly, for AS intake in tea (OR = 1.174, 95% CI 0.933~1.477, *P* = 0.172, *P*_*FDR*_ = 0.206) **(S2 Table)**. With OR of 1.020 and 1.174, we have 6% and 77% statistical power, respectively, to detect associations between AS intake in cereal and tea and T2DM.

**Fig 4:**
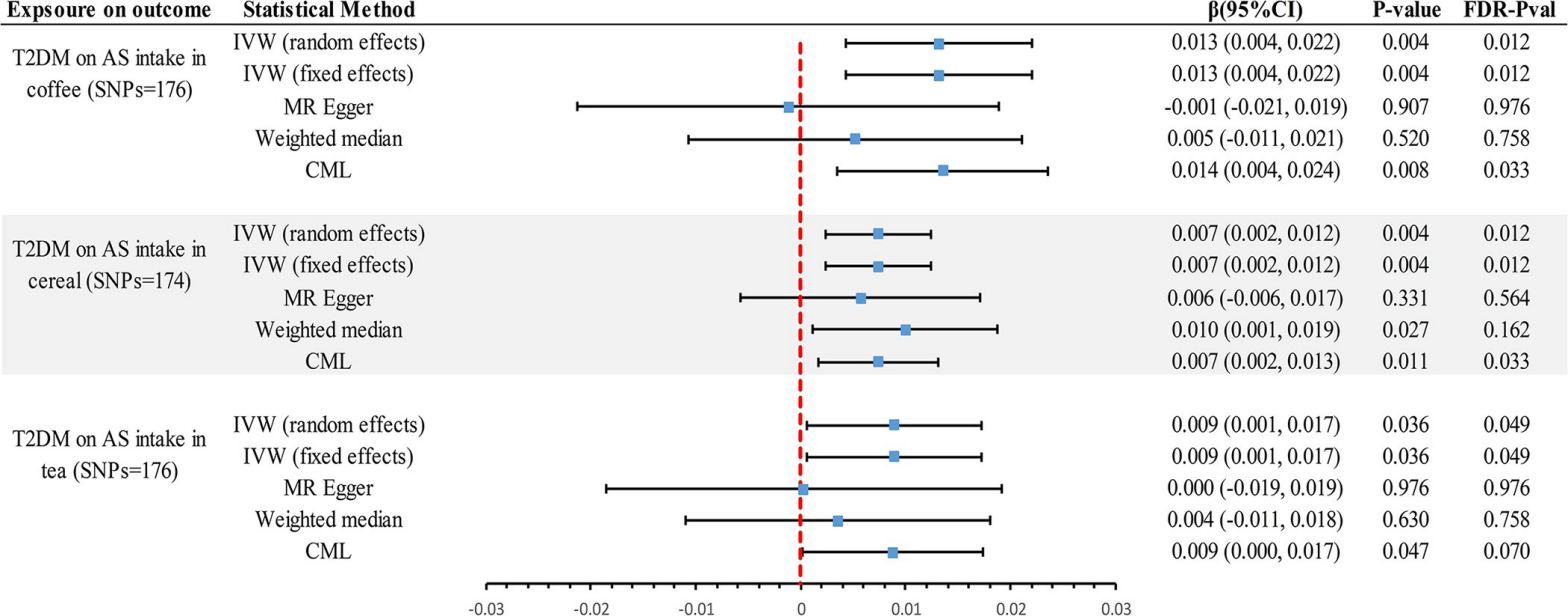
Genetically predicted causal associations of T2DM on intake of different sources of AS were assessed by different methods. IVW, Inverse-Variance-Weighted; FDR, False Discovery Rate; β, beta; CI, confidence interval; AS, Artificial sweetener; T2DM, type 2 diabetes; CML, constrained maximum likelihood.

In the reverse MR analysis utilizing the IVW method **(Fig 4)**, there is compelling evidence suggesting a positive causal relationship between T2DM and AS intake in coffee (β = 0.013, 95% CI 0.004~0.022, *P* = 0.004, *P*_*FDR*_ = 0.012), cereal (β = 0.007, 95% CI 0.002~0.012, *P* = 0.004, *P*_*FDR*_ = 0.012), and tea (β = 0.009, 95% CI 0.001~0.017, *P* = 0.036, *P*_*FDR*_ = 0.049) **(S2 Table)**. With β of 0.013, 0.007, and 0.009, we have 100% statistical power to detect associations between T2DM and AS intake in cereal, tea, and coffee, respectively.

In UVMR, the study identified AS intake in coffee with evidence for a causal relationship with T2DM that passed their statistical significance threshold (*P* < 0.05 & *P*_*FDR*_ < 0.05), and neither of these associations were significant in MVMR analyses that accounted for potential confounders (LDL-C, HDL-C, TG, HbA1c, BMI and all models) **(S3 Table)**.Further mediation MR analysis revealed that HDL-C partially mediates the causal relationship with AS intake in coffee (Mediation effect: 29.50%) and T2DM **(Table 2)**. The causal relationships among the three align with the principles of mediation MR (*P* > 0.05).

**Table 2:** Mediation analysis of the mediation effect of AS intake in coffee on T2DM via confounding factors.

Outcome	Mediator	Total effect	Direct effect	Mediation effect		
		Effect size (95% CI)	Effect size (95% CI)	Effect size (95% CI)	IE div TE(%)	*P*
T2DM	LDL-C	0.235 (0.035, 0.435)	0.239 (0.037, 0.440)	-0.004 (-0.028, 0.020)	-1.68%	0.719
	HDL-C	0.235 (0.035, 0.435)	0.166 (-0.044, 0.375)	0.069 (0.007, 0.132)	29.50%	0.026
	TG	0.235 (0.035, 0.435)	0.220 (0.016, 0.425)	0.015 (-0.027, 0.056)	6.18%	0.456
	HbA1c	0.235 (0.035, 0.435)	0.225 (0.024, 0.425)	0.010 (-0.004, 0.024)	4.22%	0.150
	BMI	0.235 (0.035, 0.435)	0.063 (-0.243, 0.369)	0.172 (-0.059, 0.403)	73.18%	0.145

BMI, body mass index; IE div TE, Indirect Effect divided by Total Effect; OR, odds ratio; CI, confidence interval; LDL-C, Low Density Lipoprotein Cholesterol; HDL-C, High Density Lipoprotein Cholesterol; TG, Triglyceride; HbA1c, Glycated Hemoglobin A1c.

A series of sensitivity analyses confirmed the robustness of the forward and reverse UVMR results **(Table 3)**. Cochran’s Q statistic suggested no heterogeneity (*P* > 0.05). MR-PRESSO detected no outliers and no evidence of horizontal pleiotropy (*P* > 0.05). MR-Egger detected no horizontal pleiotropy (*P* > 0.05). The leave-one-out analysis further validated that the causal relationship wasn’t influenced by any single SNP **(S1 Fig)**, and the funnel plot showed symmetry **(S2 Fig)**. The Steiger test indicated that all SNPs passed the test, and the direction of causality remained unchanged, further solidifying the results. The forest plots can be found in **S3 Fig**.

**Table 3:** Summary of sensitivity results.

Exposure	Outcome	MR-Egger intercept			MR-PRESSO global test			Cochrane’s Q			Steiger_test	
		Intercept	SE	*Pval*	RSS_obs_	*P*-value	*Outlier*	*Q*	*Q_df*	*Q_pval*	Direction	*Pval*
AS intake in coffee	T2DM	0.002	0.006	0.780	16.898	*0*.*361*	*NA*	13.801	13	0.388	TRUE	7.74E-65
AS intake in cereal	T2DM	-0.011	0.005	0.036	20.690	*0*.*480*	*NA*	18.773	19	0.471	TRUE	1.09E-84
AS intake in tea	T2DM	-0.003	0.007	0.719	23.803	0.150	*NA*	20.726	15	0.146	TRUE	1.12E-73
T2DM	AS intake in coffee	0.001	0.001	0.120	160.107	*0*.*827*	*NA*	158.043	175	0.816	TRUE	1.38E-112
T2DM	AS intake in tea	1.23E-04	3.88E-04	0.752	145.839	*0*.*943*	*NA*	144.285	173	0.945	TRUE	3.00E-116
T2DM	AS intake in cereal	0.001	0.001	0.317	133.698	*0*.*991*	*NA*	132.402	173	0.990	TRUE	7.25E-123

AS, Artificial sweetener; T2DM, Type 2 Diabetes Mellitus; MR, Mendelian Randomization; MR-PRESSO, MR Pleiotropy Residual Sum and Outlier.

## Discussion

This study conducted a comprehensive MR analysis to delve deeper into the genetic susceptibility linking AS intake from various sources with T2DM. The MR findings corroborated prior epidemiological studies [11–14], establishing a causal relationship between an elevated risk of AS intake in coffee and T2DM. Moreover, we identified a positive correlation between T2DM and AS intake in coffee, cereal, and tea. Further LDSC analysis revealed significant genetic correlation between the exposure and outcome phenotypes. MVMR analyses unveiled the influence of several confounding factors, while mediation MR indicated that HDL-C partially mediates the causal relationship.

Previous epidemiological research has observed a link between AS and T2DM. The findings demonstrated an association between artificial sweetener usage and the emergence of insulin resistance and T2DM among diabetic patients, leading to a heightened occurrence of obesity. However, animal studies have hinted at a negative relationship between AS and T2DM [40–42]. In contrast, thorough safety assessments have confirmed their safety [43], and reputable organizations have vouched for their safety [7]. Nevertheless, innate limitations in observational studies make it difficult to fully negate the impact of unobserved confounding variables and reverse causality. Observational studies tend to prioritize correlation over causation. By employing MR analysis, this study minimized the effects of bias and confounding factors, establishing a causal relationship between AS intake in coffee and T2DM.

This study elucidates the results through gastrointestinal reactions, insulin resistance and secretion, alterations in the microbiome, and changes in feeding behavior. Chlorogenic acid and caffeic acid, as bioactive components in coffee, are known to influence intestinal motility and gastric acid secretion, subsequently affecting food and nutrient absorption and digestion [44]. When combined with AS, these compounds could potentially influence the secretion of gut hormones. Research conducted by Jing Ma’s team suggests that AS can stimulate the secretion of GLP-1 and GIP from the intestinal endocrine cell line GLUTag, as well as GLP-1 secretion from the human L-cell line NCI-H1 [45], subsequently influencing insulin secretion and glucose homeostasis. Furthermore, intake of AS might alter the structure of the gut microbiome, resulting in gut bacterial imbalance and glucose metabolic disturbances. Consistent evidence provided by research from Jotham Suez et al. [46] indicates that AS can modify the gut microbiome to induce glucose intolerance in mice and various human subgroups, resulting in sustained hyperglycemic states. Coffee itself, with its bioactive compounds like caffeine and polyphenols, can also alter the gut flora. A review by Astrid Nehlig [47] indicates that coffee consumption mainly affects the population levels of bifidobacteria.

AS may directly or indirectly influence insulin secretion and function. Research by Cristina Bosetti and colleagues [48] suggests that AS such as saccharin can induce pancreatic cells to release insulin, resulting in short-term hyperinsulinemia. Long-term overstimulation might lead to the functional exhaustion of pancreatic cells. Caffeine can accelerate gastric emptying, temporarily increasing blood glucose and insulin resistance. A meta-analysis involving seven cohorts by Xiuqin Shi’s team supports this notion [49], suggesting that caffeine intake can reduce insulin sensitivity in healthy subjects, possibly due to interference with intracellular glucose uptake. Considering both effects, artificial sweeteners in coffee might exacerbate this burden, leading to overexertion of pancreatic cells or further glucose metabolic disruption.

Coffee, an integral part of daily life, is frequently consumed with high-sugar, high-fat foods like pastries, potentially impacting glucose absorption and metabolic rates [50]. Given the "zero-calorie" characteristic of AS, if consumers mistakenly believe that coffee with AS can offset the intake of other unhealthy foods, this could lead to an overall increase in caloric intake, thus elevating the risk of T2DM. This notion is further corroborated in the reverse MR analysis, where an increased intake of AS from various sources is associated with the T2DM. Other beverages, such as tea, might be consumed independently at other times of the day. Research by Bangde Li and colleagues suggests sweetness and coffee flavor directly influence two key sensory attributes for consumers [51]. The robust flavor of coffee, compared to tea or cereals, might necessitate more artificial sweeteners to achieve the desired sweetness. Consequently, the amount of sweetener consumed might vary, further explaining the study’s findings that AS intake from other sources doesn’t show a causal link with T2DM.

The mediation MR analysis unveils a pivotal role of HDL-C as an intermediary in the relationship between AS intake in coffee and the risk of T2DM. HDL-C, commonly termed as the ’beneficial cholesterol,’ facilitates reverse cholesterol transport by actively sequestering surplus cholesterol from peripheral tissues and conveying it to the liver for subsequent excretion [52]. AS intake in coffee may influence HDL-C levels by altering metabolism and regulating lipid metabolism through changes in the gut microbiome [53]. Ample evidence suggests that artificial sweeteners are associated with liver damage [54, 55], and liver function, in turn, impacts the synthesis and metabolism of HDL-C. Concurrently, HDL-C may affect insulin sensitivity by modulating the function of β-cells and the peripheral tissue’s glucose uptake [56]. Therefore, the mediating effect of HDL-C implies that interventions targeting the regulation of HDL-C, combined with controlling AS intake, might offer a synergistic approach for preventing or mitigating the risk of T2DM.

The study demonstrates several notable merits. Primarily, this MR analysis pioneers in establishing a causal linkage between sources of AS intake and T2DM. Furthermore, given that all SNPs utilized as IVs were identified within the European population, the probability of population stratification bias is diminished, thereby bolstering the credibility of the MR assumption. In the course of this inquiry, the application of rigorous instruments (e.g., F statistic significantly surpassing 10) serves to mitigate potential biases stemming from sample overlap [57]. However, our study is not without certain limitations. While every SNP was scrutinized, not all potential risk factors were considered. Furthermore, the selection of a relatively small number of SNPs as IVs could account for a minimal percentage of exposure variation, thereby affecting the statistical power of causal estimations. In addition, the lack of extensive disease severity and demographic information in the GWAS database, making it impossible to undertake further subgroup analyses.

### Conclusion

In summary, the MR analysis has established a causal relationship between AS intake in coffee and an elevated risk of T2DM, with HDL-C mediating a portion of this causal effect. The reverse analysis indicates a positive correlation between T2DM and artificial sweetener intake from all sources. Future MR analyses, employing larger-scale GWAS summary data and an increased number of genetic instruments, are necessary to corroborate the conclusions drawn from this study.

## Supporting information

S1 Table Power calculations for bidirectional univariable Mendelian randomization analyses. (DOCX)

S2 Table Summary of UVMR analysis results. (DOCX)

S3 Table Summary of analytical results for MVMR. (DOCX)

S1 Fig Funnel plot of instrument precision against instrumental variable estimates for each genetic variant separately for Mendelian randomization analysis of exposure on outcomes risk. (A) AS intake in coffee on T2DM (B) AS intake in tea on T2DM (C) AS intake in cereal on T2DM (D) T2DM on AS intake in coffee (E) T2DM on AS intake in tea (F) T2DM on AS intake in cereal. Solid vertical line is the (random-effect) inverse-variance weighted estimate.
(DOCX)

S2 Fig Leave-one-out plot for MR analysis of exposure on outcomes risk. (A) AS intake in coffee on T2DM (B) AS intake in tea on T2DM (C) AS intake in cereal on T2DM (D) T2DM on AS intake in coffee (E) T2DM on AS intake in tea (F) T2DM on AS intake in cereal.
(DOCX)

S3 Fig Single-SNP analysis forest plots of the effect of exposure on outcomes. (A) AS intake in coffee on T2DM (B) AS intake in tea on T2DM (C) AS intake in cereal on T2DM (D) T2DM on AS intake in coffee (E) T2DM on AS intake in tea (F) T2DM on AS intake in cereal. Point estimates represent the variant-specific ratio estimates for each SNP (in black), and the inverse-variance weighted (IVW) estimate (in orange). Horizontal lines represent 95% confidence intervals around the variant-specific ratio estimates and the IVW estimate.
(DOCX)

## References

[1] Oh MY, Kim SS, Kim IJ, Lee IK, Baek HS, Lee HW, et al. Clinical Characteristics of Diabetic Patients Transferred to Korean Referral Hospitals. Diabetes Metab J. 2014;38: 388–394. doi: 10.4093/dmj.2014.38.5.388 25349826 PMC4209353

[2] Dugani SB, Wood-Wentz CM, Mielke MM, Bailey KR, Vella A. Assessment of Disparities in Diabetes Mortality in Adults in US Rural vs Nonrural Counties, 1999–2018. JAMA Netw Open. 2022;5: e2232318. doi: 10.1001/jamanetworkopen.2022.32318 36125809 PMC9490502

[3] Saeed A, Lambojon K, Saeed H, Saleem Z, Anwer N, Aziz MM, et al. Access to Insulin Products in Pakistan: A National Scale Cross-Sectional Survey on Prices, Availability, and Affordability. Frontiers in Pharmacology. 2022;13. doi: 10.3389/fphar.2022.820621 35431962 PMC9010947

[4] Muralidhara S, Lucieri A, Dengel A, Ahmed S. Holistic multi-class classification & grading of diabetic foot ulcerations from plantar thermal images using deep learning. Health Information Science and Systems. 2022;10. doi: 10.1007/s13755-022-00194-8 36039095 PMC9418397

[5] Low B-H, Lin Y-D, Huang B-W, Chia T, Bau J-G, Huang H-Y. Impaired Microvascular Response to Muscle Stretching in Chronic Smokers With Type 2 Diabetes. Front Bioeng Biotechnol. 2020;8: 602. doi: 10.3389/fbioe.2020.00602 32596231 PMC7300253

[6] Zhang M, Chen J, Yang M, Qian C, Liu Y, Qi Y, et al. Low Doses of Sucralose Alter Fecal Microbiota in High-Fat Diet-Induced Obese Rats. Front Nutr. 2021;8: 787055. doi: 10.3389/fnut.2021.787055 35028307 PMC8751733

[7] Sugar, sweeteners and diabetes | Diabetes UK. [cited 28 May 2023]. Available: https://www.diabetes.org.uk/guide-to-diabetes/enjoy-food/carbohydrates-and-diabetes/sugar-sweeteners-and-diabetes

[8] Fajardo V, González MP, Martínez M, Samaniego-Vaesken M de L, Achón M, Úbeda N, et al. Updated Food Composition Database for Cereal-Based Gluten Free Products in Spain: Is Reformulation Moving on? Nutrients. 2020;12: 2369. doi: 10.3390/nu12082369 32784763 PMC7469026

[9] Hinkle SN, Rawal S, Bjerregaard AA, Halldorsson TI, Li M, Ley SH, et al. A prospective study of artificially sweetened beverage intake and cardiometabolic health among women at high risk. Am J Clin Nutr. 2019;110: 221–232. doi: 10.1093/ajcn/nqz094 31172169 PMC6599744

[10] Basílio M, Silva LJG, Pereira AMPT, Pena A, Lino CM. Artificial sweeteners in non-alcoholic beverages: Occurrence and exposure estimation of the Portuguese population. Food Addit Contam Part A Chem Anal Control Expo Risk Assess. 2020;37: 2040–2050. doi: 10.1080/19440049.2020.1812734 32910867

[11] Bhupathiraju SN, Pan A, Malik VS, Manson JE, Willett WC, van Dam RM, et al. Caffeinated and caffeine-free beverages and risk of type 2 diabetes. Am J Clin Nutr. 2013;97: 155–166. doi: 10.3945/ajcn.112.048603 23151535 PMC3522135

[12] InterAct Consortium, Romaguera D, Norat T, Wark PA, Vergnaud AC, Schulze MB, et al. Consumption of sweet beverages and type 2 diabetes incidence in European adults: results from EPIC-InterAct. Diabetologia. 2013;56: 1520–1530. doi: 10.1007/s00125-013-2899-8 23620057

[13] Pan A, Malik VS, Schulze MB, Manson JE, Willett WC, Hu FB. Plain-water intake and risk of type 2 diabetes in young and middle-aged women. Am J Clin Nutr. 2012;95: 1454–1460. doi: 10.3945/ajcn.111.032698 22552035 PMC3349456

[14] O’Connor L, Imamura F, Lentjes MAH, Khaw K-T, Wareham NJ, Forouhi NG. Prospective associations and population impact of sweet beverage intake and type 2 diabetes, and effects of substitutions with alternative beverages. Diabetologia. 2015;58: 1474–1483. doi: 10.1007/s00125-015-3572-1 25944371 PMC4473082

[15] Huang M, Quddus A, Stinson L, Shikany JM, Howard BV, Kutob RM, et al. Artificially sweetened beverages, sugar-sweetened beverages, plain water, and incident diabetes mellitus in postmenopausal women: the prospective Women’s Health Initiative observational study. Am J Clin Nutr. 2017;106: 614–622. doi: 10.3945/ajcn.116.145391 28659294 PMC5525115

[16] Leahy M, Ratliff JC, Riedt CS, Fulgoni VL. Consumption of Low-Calorie Sweetened Beverages Compared to Water Is Associated with Reduced Intake of Carbohydrates and Sugar, with No Adverse Relationships to Glycemic Responses: Results from the 2001–2012 National Health and Nutrition Examination Surveys. Nutrients. 2017;9: 928. doi: 10.3390/nu9090928 28837084 PMC5622688

[17] Ma J, Jacques PF, Meigs JB, Fox CS, Rogers GT, Smith CE, et al. Sugar-Sweetened Beverage but Not Diet Soda Consumption Is Positively Associated with Progression of Insulin Resistance and Prediabetes. J Nutr. 2016;146: 2544–2550. doi: 10.3945/jn.116.234047 27934644 PMC5118762

[18] Lawlor DA. Commentary: Two-sample Mendelian randomization: opportunities and challenges. Int J Epidemiol. 2016;45: 908–915. doi: 10.1093/ije/dyw127 27427429 PMC5005949

[19] Lawlor DA, Harbord RM, Sterne JAC, Timpson N, Davey Smith G. Mendelian randomization: Using genes as instruments for making causal inferences in epidemiology. Statistics in Medicine. 2008;27: 1133–1163. doi: 10.1002/sim.3034 17886233

[20] Sudlow C, Gallacher J, Allen N, Beral V, Burton P, Danesh J, et al. UK biobank: an open access resource for identifying the causes of a wide range of complex diseases of middle and old age. PLoS Med. 2015;12: e1001779. doi: 10.1371/journal.pmed.1001779 25826379 PMC4380465

[21] Chen J, Ruan X, Yuan S, Deng M, Zhang H, Sun J, et al. Antioxidants, minerals and vitamins in relation to Crohn’s disease and ulcerative colitis: A Mendelian randomization study. Aliment Pharmacol Ther. 2023;57: 399–408. doi: 10.1111/apt.17392 36645152 PMC11497233

[22] Bowden J, Del Greco M F, Minelli C, Davey Smith G, Sheehan NA, Thompson JR. Assessing the suitability of summary data for two-sample Mendelian randomization analyses using MR-Egger regression: the role of the I2 statistic. Int J Epidemiol. 2016;45: 1961–1974. doi: 10.1093/ije/dyw220 27616674 PMC5446088

[23] Hemani G, Tilling K, Davey Smith G. Orienting the causal relationship between imprecisely measured traits using GWAS summary data. PLoS Genet. 2017;13: e1007081. doi: 10.1371/journal.pgen.1007081 29149188 PMC5711033

[24] Mahajan A, Spracklen CN, Zhang W, Ng MCY, Petty LE, Kitajima H, et al. Multi-ancestry genetic study of type 2 diabetes highlights the power of diverse populations for discovery and translation. Nat Genet. 2022;54: 560–572. doi: 10.1038/s41588-022-01058-3 35551307 PMC9179018

[25] Yengo L, Sidorenko J, Kemper KE, Zheng Z, Wood AR, Weedon MN, et al. Meta-analysis of genome-wide association studies for height and body mass index in ∼700000 individuals of European ancestry. Hum Mol Genet. 2018;27: 3641–3649. doi: 10.1093/hmg/ddy271 30124842 PMC6488973

[26] Soranzo N, Sanna S, Wheeler E, Gieger C, Radke D, Dupuis J, et al. Common variants at 10 genomic loci influence hemoglobin A₁(C) levels via glycemic and nonglycemic pathways. Diabetes. 2010;59: 3229–3239. doi: 10.2337/db10-0502 20858683 PMC2992787

[27] Willer CJ, Schmidt EM, Sengupta S, Peloso GM, Gustafsson S, Kanoni S, et al. Discovery and refinement of loci associated with lipid levels. Nat Genet. 2013;45: 1274–1283. doi: 10.1038/ng.2797 24097068 PMC3838666

[28] Hemani G, Zheng J, Elsworth B, Wade KH, Haberland V, Baird D, et al. The MR-Base platform supports systematic causal inference across the human phenome. Elife. 2018;7: e34408. doi: 10.7554/eLife.34408 29846171 PMC5976434

[29] Bowden J, Davey Smith G, Haycock PC, Burgess S. Consistent Estimation in Mendelian Randomization with Some Invalid Instruments Using a Weighted Median Estimator. Genet Epidemiol. 2016;40: 304–314. doi: 10.1002/gepi.21965 27061298 PMC4849733

[30] Zhang L, Mukherjee B, Ghosh M, Gruber S, Moreno V. Accounting for error due to misclassification of exposures in case-control studies of gene-environment interaction. Stat Med. 2008;27: 2756–2783. doi: 10.1002/sim.3044 17879261

[31] Burgess S, Thompson SG. Multivariable Mendelian randomization: the use of pleiotropic genetic variants to estimate causal effects. Am J Epidemiol. 2015;181: 251–260. doi: 10.1093/aje/kwu283 25632051 PMC4325677

[32] Tobin MD, Minelli C, Burton PR, Thompson JR. Commentary: development of Mendelian randomization: from hypothesis test to “Mendelian deconfounding.” Int J Epidemiol. 2004;33: 26–29. doi: 10.1093/ije/dyh016 15075142

[33] Kulinskaya E, Dollinger MB, Bjørkestøl K. On the moments of Cochran’s Q statistic under the null hypothesis, with application to the meta-analysis of risk difference. Res Synth Methods. 2020;11: 920. doi: 10.1002/jrsm.1446 33190421

[34] Burgess S, Thompson SG. Interpreting findings from Mendelian randomization using the MR-Egger method. Eur J Epidemiol. 2017;32: 377–389. doi: 10.1007/s10654-017-0255-x 28527048 PMC5506233

[35] Wu F, Huang Y, Hu J, Shao Z. Mendelian randomization study of inflammatory bowel disease and bone mineral density. BMC Med. 2020;18: 312. doi: 10.1186/s12916-020-01778-5 33167994 PMC7654011

[36] Verbanck M, Chen C-Y, Neale B, Do R. Detection of widespread horizontal pleiotropy in causal relationships inferred from Mendelian randomization between complex traits and diseases. Nat Genet. 2018;50: 693–698. doi: 10.1038/s41588-018-0099-7 29686387 PMC6083837

[37] Cheng H, Garrick DJ, Fernando RL. Efficient strategies for leave-one-out cross validation for genomic best linear unbiased prediction. J Anim Sci Biotechnol. 2017;8: 38. doi: 10.1186/s40104-017-0164-6 28469846 PMC5414316

[38] Guan W, Steffen BT, Lemaitre RN, Wu JHY, Tanaka T, Manichaikul A, et al. Genome-Wide Association Study of Plasma N6 Polyunsaturated Fatty Acids within the CHARGE Consortium. Circ Cardiovasc Genet. 2014;7: 321–331. doi: 10.1161/CIRCGENETICS.113.000208 24823311 PMC4123862

[39] Brion M-JA, Shakhbazov K, Visscher PM. Calculating statistical power in Mendelian randomization studies. Int J Epidemiol. 2013;42: 1497–1501. doi: 10.1093/ije/dyt179 24159078 PMC3807619

[40] Davidson TL, Sample CH, Swithers SE. An application of Pavlovian principles to the problems of obesity and cognitive decline. Neurobiol Learn Mem. 2014;108: 172–184. doi: 10.1016/j.nlm.2013.07.014 23887140 PMC3899105

[41] Deutsch R. Conditioned hypoglycemia: a mechanism for saccharin-induced sensitivity to insulin in the rat. J Comp Physiol Psychol. 1974;86: 350–358. doi: 10.1037/h0035948 4811558

[42] Bian X, Chi L, Gao B, Tu P, Ru H, Lu K. The artificial sweetener acesulfame potassium affects the gut microbiome and body weight gain in CD-1 mice. PLoS One. 2017;12: e0178426. doi: 10.1371/journal.pone.0178426 28594855 PMC5464538

[43] Atallah AA, Morsy OM, Abbas W, Khater E-SG. Microstructural, Physicochemical, Microbiological, and Organoleptic Characteristics of Sugar- and Fat-Free Ice Cream from Buffalo Milk. Foods. 2022;11: 490. doi: 10.3390/foods11030490 35159640 PMC8833986

[44] Tajik N, Tajik M, Mack I, Enck P. The potential effects of chlorogenic acid, the main phenolic components in coffee, on health: a comprehensive review of the literature. Eur J Nutr. 2017;56: 2215–2244. doi: 10.1007/s00394-017-1379-1 28391515

[45] Ma J, Chang J, Checklin HL, Young RL, Jones KL, Horowitz M, et al. Effect of the artificial sweetener, sucralose, on small intestinal glucose absorption in healthy human subjects. British Journal of Nutrition. 2010;104: 803–806. doi: 10.1017/S0007114510001327 20420761

[46] Suez J, Korem T, Zilberman-Schapira G, Segal E, Elinav E. Non-caloric artificial sweeteners and the microbiome: findings and challenges. Gut Microbes. 2015;6: 149–155. doi: 10.1080/19490976.2015.1017700 25831243 PMC4615743

[47] Nehlig A. Effects of Coffee on the Gastro-Intestinal Tract: A Narrative Review and Literature Update. Nutrients. 2022;14: 399. doi: 10.3390/nu14020399 35057580 PMC8778943

[48] Bosetti C, Gallus S, Talamini R, Montella M, Franceschi S, Negri E, et al. Artificial Sweeteners and the Risk of Gastric, Pancreatic, and Endometrial Cancers in Italy. Cancer Epidemiology, Biomarkers & Prevention. 2009;18: 2235–2238. doi: 10.1158/1055-9965.EPI-09-0365 19661082

[49] Shi X, Xue W, Liang S, Zhao J, Zhang X. Acute caffeine ingestion reduces insulin sensitivity in healthy subjects: a systematic review and meta-analysis. Nutr J. 2016;15: 103. doi: 10.1186/s12937-016-0220-7 28031026 PMC5192567

[50] Radhika G, Sathya RM, Ganesan A, Saroja R, Vijayalakshmi P, Sudha V, et al. Dietary profile of urban adult population in South India in the context of chronic disease epidemiology (CURES-68). Public Health Nutr. 2011;14: 591–598. doi: 10.1017/S136898001000203X 20701818

[51] Li B, Hayes JE, Ziegler GR. Just-About-Right and ideal scaling provide similar insights into the influence of sensory attributes on liking. Food Qual Prefer. 2014;37: 71–78. doi: 10.1016/j.foodqual.2014.04.019 25061258 PMC4104712

[52] Ouimet M, Barrett TJ, Fisher EA. HDL and Reverse Cholesterol Transport. Circ Res. 2019;124: 1505–1518. doi: 10.1161/CIRCRESAHA.119.312617 31071007 PMC6813799

[53] Suez J, Korem T, Zeevi D, Zilberman-Schapira G, Thaiss CA, Maza O, et al. Artificial sweeteners induce glucose intolerance by altering the gut microbiota. Nature. 2014;514: 181–186. doi: 10.1038/nature13793 25231862

[54] Shearer J, Swithers S. Artificial sweeteners and metabolic dysregulation: Lessons learned from agriculture and the laboratory. Reviews in Endocrine and Metabolic Disorders. 2016;17. doi: 10.1007/s11154-016-9372-1 27387506

[55] Abbas T, Murad W. Do artificial sweeteners increase the risk of non-alcoholic fatty liver disease (NAFLD)? EXCLI Journal. 2020;19. doi: 10.17179/excli2020-2745 33088253 PMC7573173

[56] Ağagündüz D, Icer MA, Yesildemir O, Koçak T, Kocyigit E, Capasso R. The roles of dietary lipids and lipidomics in gut-brain axis in type 2 diabetes mellitus. Journal of Translational Medicine. 2023;21: 240. doi: 10.1186/s12967-023-04088-5 37009872 PMC10068184

[57] Pierce BL, Burgess S. Efficient design for Mendelian randomization studies: subsample and 2-sample instrumental variable estimators. Am J Epidemiol. 2013;178: 1177–1184. doi: 10.1093/aje/kwt084 23863760 PMC3783091

