# Supplementary material for: Associations between artificial sweetener intake from cereals, coffee, and tea and the risk of type 2 diabetes mellitus: A genetic correlation, mediation, and mendelian randomization analysis
Source: PLoS One. 2024 Feb 7;19(2):e0287496. doi: 10.1371/journal.pone.0287496 (PMC10849235; doi:10.1371/journal.pone.0287496)
Supplement: S1 Table — (DOCX) [file pone.0287496.s001.docx]

| **Supplementary file -Table 1.** Power calculations for bidirectional univariable Mendelian randomization analyses. | | | | | |
| --- | --- | --- | --- | --- | --- |
| **Exposure** | **Outcome** | **SNPs** | **Proportion of variance in the exposure explained by the instrument (*R^2^*)** | **F-statistic** | **Power-statistic** |
| Artificial sweetener intake in coffee | T2DM (DIAGRAM) | 14 | 0.002 | 26.176 | 98% |
| Artificial sweetener intake in cereal | T2DM (DIAGRAM) | 20 | 0.001 | 23.583 | 6% |
| Artificial sweetener intake in tea | T2DM (DIAGRAM) | 16 | 0.002 | 26.200 | 77% |
| T2DM (DIAGRAM) | Artificial sweetener intake in coffee | 176 | 0.285 | 77.519 | 100% |
| T2DM (DIAGRAM) | Artificial sweetener intake in cereal | 174 | 0.282 | 77.690 | 100% |
| T2DM (DIAGRAM) | Artificial sweetener intake in tea | 176 | 0.283 | 77.834 | 100% |
