# Supplementary material for: Associations between artificial sweetener intake from cereals, coffee, and tea and the risk of type 2 diabetes mellitus: A genetic correlation, mediation, and mendelian randomization analysis
Source: PLoS One. 2024 Feb 7;19(2):e0287496. doi: 10.1371/journal.pone.0287496 (PMC10849235; doi:10.1371/journal.pone.0287496)
Supplement: S2 Table — (DOCX) [file pone.0287496.s002.docx]

| **Supplementary file-Table 2 .** Summary of UVMR analysis results | | | | | | | | | | | |  | |
| --- | --- | --- | --- | --- | --- | --- | --- | --- | --- | --- | --- | --- | --- |
| **Methods** |  | **AS intake in coffee on T2DM** | | | | |  | **T2DM on AS intake in coffee** | | | | | |
|  |  | **OR** | **or_lci95** | **or_uci95** | **P-value** | **FDR-Pval** |  | **β** | **lo_ci** | **up_ci** | **P-value** | **FDR-Pval** |  |
| Inverse variance weighted (random effects) |  | 1.265 | 1.035 | 1.545 | 0.021 | 0.042 |  | 0.013 | 0.004 | 0.022 | 0.004 | 0.012 |  |
| Inverse variance weighted (fixed effects) |  | 1.265 | 1.042 | 1.535 | 0.018 | 0.036 |  | 0.013 | 0.004 | 0.022 | 0.004 | 0.012 |  |
| MR Egger |  | 1.204 | 0.810 | 1.789 | 0.376 | 0.564 |  | -0.001 | -0.021 | 0.019 | 0.907 | 0.976 |  |
| Weighted median |  | 1.149 | 0.883 | 1.495 | 0.300 | 0.758 |  | 0.005 | -0.011 | 0.021 | 0.520 | 0.758 |  |
| Constrained maximum likelihood |  | 1.276 | 1.035 | 1.574 | 0.022 | 0.044 |  | 0.014 | 0.004 | 0.024 | 0.008 | 0.033 |  |
| **Methods** |  | **AS intake in cereal on T2DM** | | | | |  | **T2DM on AS intake in cereal** | | | | | |
|  |  | **OR** | **or_lci95** | **or_uci95** | **P-value** | **FDR-Pval** |  | **β** | **lo_ci** | **up_ci** | **P-value** | **FDR-Pval** |  |
| Inverse variance weighted (random effects) |  | 1.020 | 0.755 | 1.378 | 0.897 | 0.897 |  | 0.007 | 0.002 | 0.012 | 0.004 | 0.012 |  |
| Inverse variance weighted (fixed effects) |  | 1.020 | 0.755 | 1.378 | 0.897 | 0.897 |  | 0.007 | 0.002 | 0.012 | 0.004 | 0.012 |  |
| MR Egger |  | 1.837 | 1.017 | 3.317 | 0.059 | 0.354 |  | 0.006 | -0.006 | 0.017 | 0.331 | 0.564 |  |
| Weighted median |  | 1.111 | 0.722 | 1.710 | 0.632 | 0.758 |  | 0.010 | 0.001 | 0.019 | 0.027 | 0.162 |  |
| Constrained maximum likelihood |  | 1.003 | 0.745 | 1.350 | 0.985 | 0.985 |  | 0.007 | 0.002 | 0.013 | 0.011 | 0.033 |  |
| **Methods** |  | **AS intake in tea on T2DM** | | | | |  | **T2DM on AS intake in tea** | | | | | |
|  |  | **OR** | **or_lci95** | **or_uci95** | **P-value** | **FDR-Pval** |  | **β** | **lo_ci** | **up_ci** | **P-value** | **FDR-Pval** |  |
| Inverse variance weighted (random effects) |  | 1.174 | 0.933 | 1.477 | 0.172 | 0.206 |  | 0.009 | 0.001 | 0.017 | 0.036 | 0.049 |  |
| Inverse variance weighted (fixed effects) |  | 1.174 | 0.965 | 1.428 | 0.108 | 0.129 |  | 0.009 | 0.001 | 0.017 | 0.036 | 0.049 |  |
| MR Egger |  | 1.282 | 0.757 | 2.169 | 0.371 | 0.564 |  | 0.000 | -0.019 | 0.019 | 0.976 | 0.976 |  |
| Weighted median |  | 0.970 | 0.726 | 1.296 | 0.836 | 0.836 |  | 0.004 | -0.011 | 0.018 | 0.630 | 0.758 |  |
| Constrained maximum likelihood |  | 1.160 | 0.921 | 1.462 | 0.208 | 0.249 |  | 0.009 | 0.000 | 0.017 | 0.047 | 0.07 |  |
