# Supplementary material for: Associations between artificial sweetener intake from cereals, coffee, and tea and the risk of type 2 diabetes mellitus: A genetic correlation, mediation, and mendelian randomization analysis
Source: PLoS One. 2024 Feb 7;19(2):e0287496. doi: 10.1371/journal.pone.0287496 (PMC10849235; doi:10.1371/journal.pone.0287496)
Supplement: S3 Table — (DOCX) [file pone.0287496.s003.docx]

| **Supplementary file -Table 3.**Summary of analytical results for MVMR | | | | | |
| --- | --- | --- | --- | --- | --- |
| MVMR models | SNP | beta | se | pval | OR(95%CI) |
| LDL-C | 80 | 0.221 | 0.450 | 0.623 | 1.247 (0.517, 3.011) |
| HDL-C | 86 | 0.353 | 0.621 | 0.570 | 1.423 (0.421, 4.811) |
| TG | 56 | 0.269 | 0.854 | 0.753 | 1.308 (0.245, 6.975) |
| HbA1c | 207 | 0.382 | 0.239 | 0.109 | 1.465 (0.918, 2.339) |
| BMI | 476 | 0.149 | 0.246 | 0.546 | 1.160 (0.716, 1.879) |
| ALL models | 390 | 0.315 | 0.311 | 0.312 | 1.370 (0.745, 2.520) |
| LDL-C, Low Density Lipoprotein Cholesterol; HDL-C, High Density Lipoprotein Cholesterol; TG, Triglyceride; HbA1c, Glycated Hemoglobin A1c; BMI, body mass index. | | | | | |
