# Supplementary material for: Associations between artificial sweetener intake from cereals, coffee, and tea and the risk of type 2 diabetes mellitus: A genetic correlation, mediation, and mendelian randomization analysis
Source: PLoS One. 2024 Feb 7;19(2):e0287496. doi: 10.1371/journal.pone.0287496 (PMC10849235; doi:10.1371/journal.pone.0287496)
Supplement: S2 Fig — (A) AS intake in coffee on T2DM (B) AS intake in tea on T2DM (C) AS intake in cereal on T2DM (D) T2DM on AS intake in coffee (E) T2DM on AS intake in tea (F) T2DM on AS intake in cereal. (DOCX) [file pone.0287496.s005.docx]

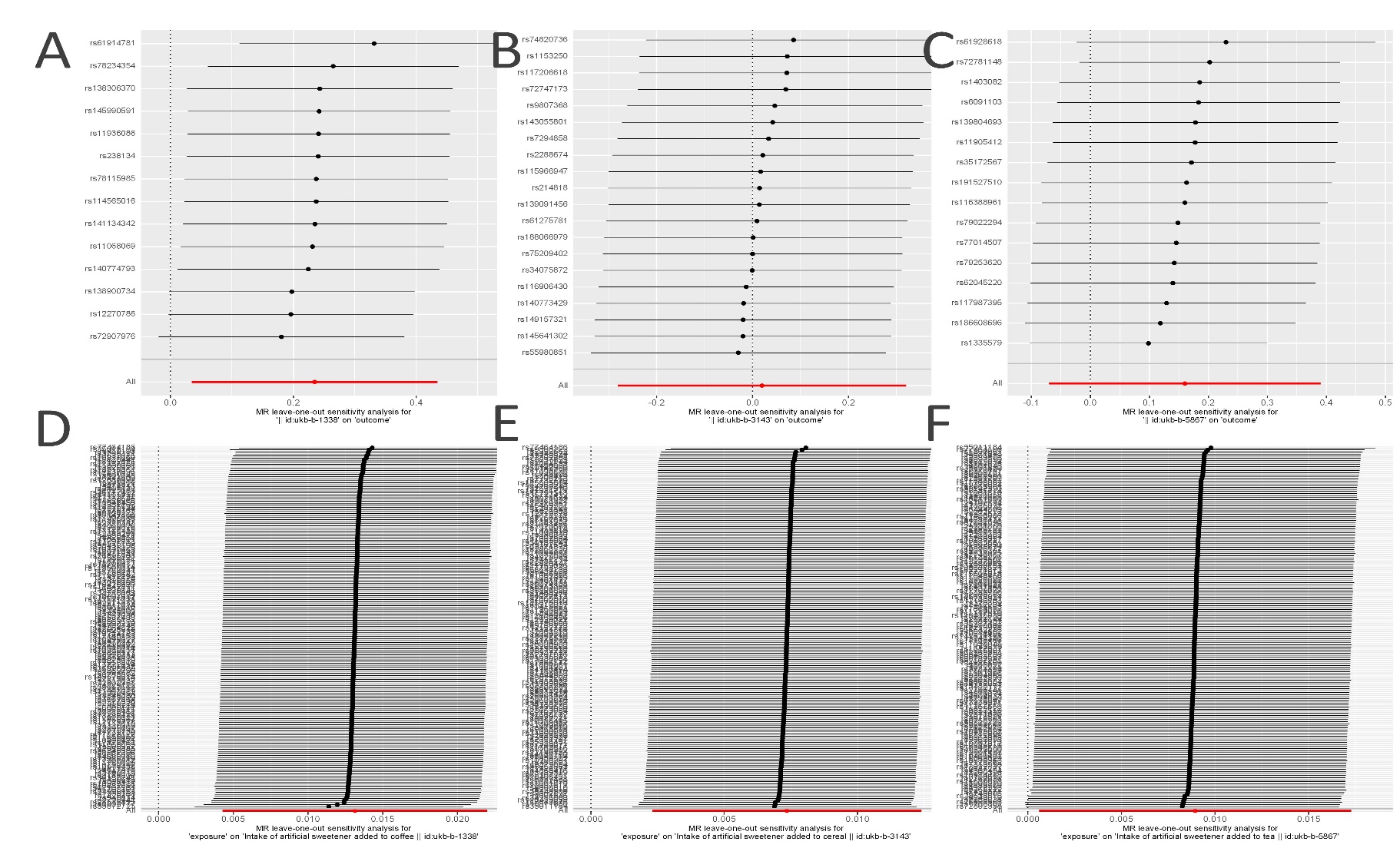


**Supplementary Fig. 2** Leave-one-out plot for MR analysis of exposure on outcomes risk. (A) AS intake in coffee on T2DM (B) AS intake in tea on T2DM (C) AS intake in cereal on T2DM (D) T2DM on AS intake in coffee (E) T2DM on AS intake in tea (F) T2DM on AS intake in cereal.
