# Supplementary material for: Associations between artificial sweetener intake from cereals, coffee, and tea and the risk of type 2 diabetes mellitus: A genetic correlation, mediation, and mendelian randomization analysis
Source: PLoS One. 2024 Feb 7;19(2):e0287496. doi: 10.1371/journal.pone.0287496 (PMC10849235; doi:10.1371/journal.pone.0287496)
Supplement: S3 Fig — (A) AS intake in coffee on T2DM (B) AS intake in tea on T2DM (C) AS intake in cereal on T2DM (D) T2DM on AS intake in coffee (E) T2DM on AS intake in tea (F) T2DM on AS intake in cereal. Point estimates represent the variant-specific ratio estimates for each SNP (in black), and the inverse-variance weighted (IVW) estimate (in orange). Horizontal lines represent 95% confidence intervals around the variant-specific ratio estimates and the IVW estimate. (DOCX) [file pone.0287496.s006.docx]

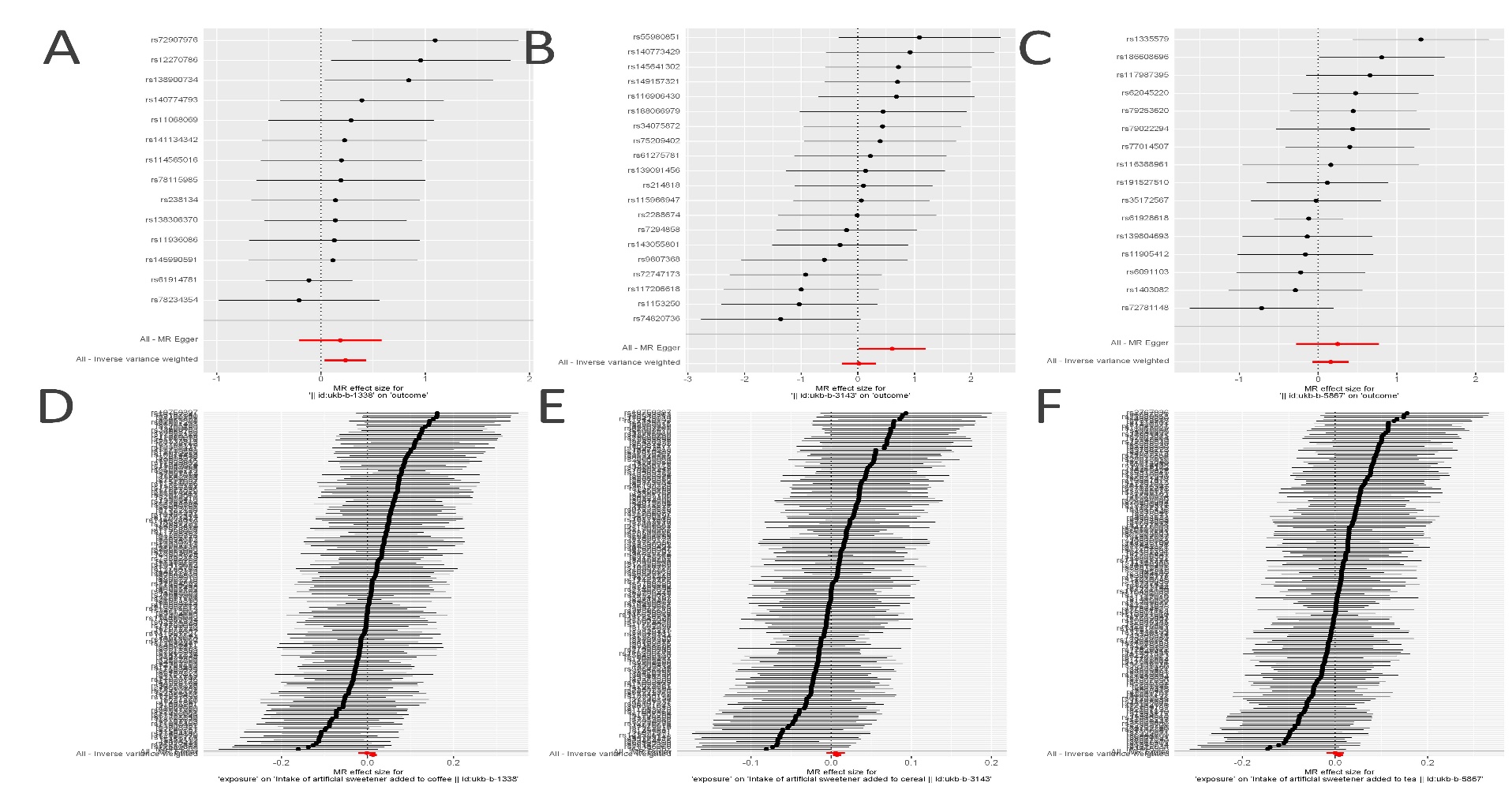


**Supplementary Fig. 3** Single-SNP analysis forest plots of the effect of exposure on outcomes. (A) AS intake in coffee on T2DM (B) AS intake in tea on T2DM (C) AS intake in cereal on T2DM (D) T2DM on AS intake in coffee (E) T2DM on AS intake in tea (F) T2DM on AS intake in cereal. Point estimates represent the variant-specific ratio estimates for each SNP (in black), and the inverse-variance weighted (IVW) estimate (in orange). Horizontal lines represent 95% confidence intervals around the variant-specific ratio estimates and the IVW estimate.
